# 2714. Invasive Fungal Disease in Lung Transplant Recipients: Incidence, Risk Factors, and Outcomes

**DOI:** 10.1093/ofid/ofad500.2325

**Published:** 2023-11-27

**Authors:** Victor Kovac, Hilary J Goldberg, Andy J Kim, Mary-Ruth M Joyce, Hari R Mallidi, Antonio Coppolino, Keri Townsend, Tany Thaniyavarn, John C Kennedy, Stefi F Lee, Valerie Durney, Alexis Liakos, Courtney E Harris, Jessica S Little, Amy C Sherman, Nicolas C Issa, Lindsey R Baden, Nirmal S Sharma, Ann E Woolley

**Affiliations:** Brigham & Women's Hospital, BROOKLINE, Massachusetts; Brigham and Women's Hospital, Boston, Massachusetts; Brigham and Women's Hospital, Boston, Massachusetts; Brigham and Women's Hospital, Boston, Massachusetts; Brigham and Women's Hospital, Boston, Massachusetts; Brigham and Women's Hospital, Boston, Massachusetts; Brigham & Women's Hospital, BROOKLINE, Massachusetts; Brigham and Women's Hospital, Boston, Massachusetts; Brigham and Women's Hospital, Boston, Massachusetts; Brigham and Women's Hospital, Boston, Massachusetts; Brigham & Women's Hospital, BROOKLINE, Massachusetts; Brigham & Women's Hospital/Dana-Farber Cancer Institute, boston, Massachusetts; Brigham and Women’s Hospital, Boston, Massachusetts; Brigham and Women's Hospital, Boston, Massachusetts; Brigham and Women's Hospital, Boston, Massachusetts; Brigham & Women's Hospital, BROOKLINE, Massachusetts; Brigham and Women's Hospital, Boston, Massachusetts; Brigham and Women's Hospital, Boston, Massachusetts; Brigham and Women's Hospital, Boston, Massachusetts

## Abstract

**Background:**

Invasive fungal disease (IFD) is common after lung transplant and is associated with increased morbidity and mortality. Antifungal prophylaxis is a common preventative strategy against IFD in lung transplant recipients (LTRs). We assessed the incidence of IFD in the first 3-years post-transplant, diagnosed by EORTC/MSG criteria, risk factors for developing IFD, and outcomes of LTRs stratified by development of IFD at a single center that does not use universal antifungal prophylaxis post-lung transplant.

Table 1

**Figure:**
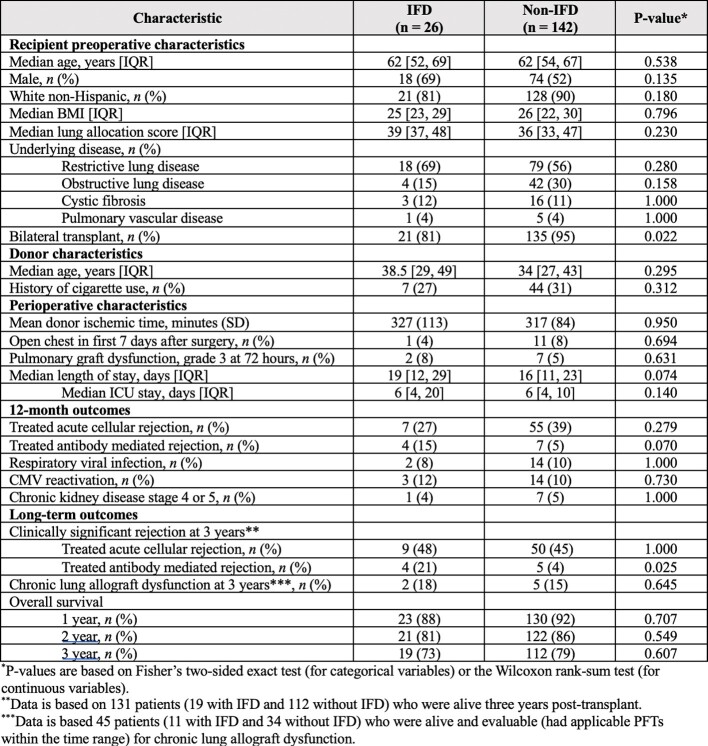

Preoperative and perioperative characteristics and clinical outcomes of lung transplant recipients stratified by invasive fungal disease (IFD) status

**Methods:**

A single-center, retrospective study of lung transplant recipients between January 2017 and December 2019 was performed with 3-year follow-up. Donor and recipient preoperative characteristics, microbiologic and radiographic data, perioperative characteristics, and 1- and 3-year outcomes were adjudicated and analyzed.

**Results:**

In this 3-year study period, 168 patients underwent lung transplant. 26 (15%) developed IFD; 23 (14%) were proven or probable, and 3 (1%) were possible [Table 1]. 21 (81%) had evidence of pulmonary infection and 17 (65%) were mold infections with Aspergillus being the predominant pathogen. 4 (15%) were Candida. 81% (21/26) of patients who developed IFD were recipients of bilateral lung transplants compared to 95% (135/142) of patients who did not develop IFD (p = 0.022). Other donor/recipient characteristics and 12-month outcomes between patients who did vs did not develop IFD were similar. 21% of patients (4/19) who developed IFD had developed antibody-mediated rejection (AMR) by three years post-transplant compared to only 4% of patients without IFD (5/112) (p = 0.025). The incidence of acute cellular rejection, chronic allograft rejection, and overall survival at three years were similar.

**Conclusion:**

Despite the lack of universal antifungal prophylaxis in the first 90 days post-transplant, we observed a low incidence of IFD in LTRs at our center in this 3-year cohort. Other than receipt of a single vs bilateral lung transplant, there were no significant differences in preoperative or perioperative recipient characteristics. Other than an increased proportion of patients who had AMR, patients who developed IFD had similar overall outcomes to those who did not have IFD, including overall survival at 1, 2, and 3 years.

**Disclosures:**

**Nicolas C. Issa, MD**, AiCuris: Grant/Research Support|Astellas: Grant/Research Support|Boehringer Ingelheim: Advisor/Consultant|Fujifilm: Grant/Research Support|GSK: Grant/Research Support|Merck: Grant/Research Support|Moderna: Grant/Research Support

